# Impact of free thyroxine levels and other clinical factors on bare metal stent restenosis

**DOI:** 10.1590/2359-3997000000197

**Published:** 2016-08-23

**Authors:** Uğur Canpolat, Osman Turak, Fırat Özcan, Fatih Öksüz, Mehmet Ali Mendi, Çağrı Yayla, Sinan Aydoğdu

**Affiliations:** 1 Türkiye Yüksek Ihtisas Training and Research Hospital Cardiology Clinic Ankara Turkey Türkiye Yüksek Ihtisas Training and Research Hospital, Cardiology Clinic, Ankara, Turkey

**Keywords:** Serum free thyroxine, bare metal stent, restenosis

## Abstract

**Objective:**

Thyroid hormones have both direct and indirect effects on thermogenesis such as modulating vascular smooth muscle cell proliferation. However, the influence of more subtle changes in thyroid hormones on coronary atherosclerosis remains a matter of speculation. Smooth muscle cells play a crucial role in the pathogenesis of in-stent restenosis (ISR). However, the relationship between free thyroxine (fT4) and ISR has not been studied. In the present study, we aimed to assess the role of preprocedural serum fT4 level on the development of ISR in patients undergoing coronary bare metal stent (BMS) implantation.

**Materials and methods:**

We enrolled and analyzed clinical, biochemical, and angiographic data from 705 consecutive patients without a history of primary thyroid disease [mean age 60.3 ± 9.3 years, 505 (72%) male]; all patients had undergone BMS implantation and further control coronary angiography owing to stable or unstable angina pectoris. Patients were divided into 3 tertiles based on preprocedural serum fT4 levels.

**Results:**

ISR was observed in 53 (23%) patients in the lowest tertile, 82 (35%) patients in the second tertile, and 107 (46%) patients in the highest fT4 tertile (p < 0.001). Using multiple logistic regression analysis, five characteristics emerged as independent predictors of ISR: diabetes mellitus, smoking, HDL-cholesterol, stent length, and preprocedural serum fT4 level. In receiver operating characteristics curve analysis, fT4 level > 1.23 mg/dL had 70% sensitivity and 73% specificity (AUC: 0.75, p < 0.001) in predicting ISR.

**Conclusion:**

Higher preprocedural serum fT4 is a powerful and independent predictor of BMS restenosis in patients with stable and unstable angina pectoris.

## INTRODUCTION

Thyroid hormones (TH) falling within and outside of the normal range have both direct and indirect effects on atherogenesis (1). While TH indirectly modifies atherosclerotic risk factors such as lipid profile (2,3) and blood pressure (4), direct effects act via vascular smooth muscle cells, altering vascular tone (5), angiotensin-II type 1 receptor modulating the proliferation of vascular smooth muscle cells (6), upregulation of basic fibroblast growth factor causing enhanced angiogenesis (7), modulating the maturation and functioning of macrophages (8), and acting on the renin-angiotensin system (9,10). However, clinical studies yield conflicting results and mechanistic explanations remain elusive (11-13).

In-stent restenosis (ISR) continues to be a major pitfall for interventional cardiologists, and numerous efforts have been made to predict or resolve this important problem (14,15). Various reactions following percutaneous coronary intervention (PCI) mediated vascular damage occur in sequence including inflammation, granulation, extracellular matrix remodeling, and vascular smooth muscle cell proliferation and migration, which result in neointimal hyperplasia and restenosis (16,17). Besides procedural factors, we hypothesize that patient-related factors such as preprocedural TH status may also be important in the development of ISR. Although previous evidence has shown several roles for THs in angiogenesis, macrophage functioning, and vascular smooth muscle cell function and proliferation, to the best of our knowledge, there has been no study investigating any possible association between pre-procedural TH levels and ISR. Here, we aimed to evaluate the association of TH levels before successful bare metal stent (BMS) implantation in predicting ISR in patients with stable and unstable angina pectoris.

## MATERIALS AND METHODS

We retrieved clinical, laboratory, and angiographic data of consecutive patients who had undergone successful BMS implantation between January 2008 and August 2010 at our tertiary center hospital. The inclusion criteria were as follows: (a) patients with stable or unstable angina, (b) coronary angiography showing *de-novo* lesions without a history of the previous PCI, and (c) patients who received coronary BMS implantation. Patient data was accessed retrospectively with the time of interest being the point at which the patients underwent BMS implantation after control coronary angiography was performed because of clinical indications that included anginal symptoms and abnormal non-invasive test results (either treadmill exercise test or myocardial perfusion scintigraphy), thus recalling clinical, angiographic, and laboratory characteristics at that time. As such, we were able to collect the data from 772 patients. Patients with unstable angina pectoris were identified according to the definition of Braunwald (18). As part of our preprocedural protocol, thyroid hormone levels were obtained prior to coronary angiography for all patients. Patients were excluded from analysis if they had been using thyroid replacement therapy or anti-thyroid drugs (n = 8), showed clinical evidence of neoplastic diseases (n = 2), heart failure [left ventricular ejection fraction (LVEF) of < 50%] (n = 18), renal dysfunction [estimated glomerular filtration rate (eGFR) of < 90 mL/min/1.73 m^2^] (n = 20), hepatic and hemolytic disorders (n = 1), chronic inflammatory disease (n = 3), used therapy with amiodarone (n = 7), or had any active infectious disease (n = 2) or sepsis (n = 1), alcohol consumption (n = 3), and patients having major adverse event during follow-up (n = 2), leaving 705 patients to be included in the study for analysis.

Clinical and demographic characteristics of patients encompassing age, gender, history of arterial hypertension, diabetes mellitus, smoking, family history of coronary artery disease, LVEF, and medications used were noted. In addition, serum levels of fasting blood glucose, serum creatinine, and lipid panel including total cholesterol, low-density lipoprotein cholesterol, high-density lipoprotein cholesterol, and triglyceride levels were recorded. Our study was in compliance with the principles outlined in the Declaration of Helsinki and approved by Institutional Ethics Committee.

All laboratory data were obtained from venous blood samples up to 6 h before stent implantation. Complete blood count and serum lipid/biochemistry panel were measured according to standard methods. Measurement of thyroid-stimulating hormone (TSH) (reference range, 0.34–5.6 µIU/mL), free triiodothyronine (fT3) (reference range, 2.5–3.9 pg/mL), and free thyroxine (fT4) (reference range, 0.54–1.24 ng/dL) levels was performed with a Beckman Coulter DXI 800 auto analyzer (Beckman Coulter, Brea, CA, USA) using the chemiluminescence method. Intra assay coefficients of variations were as follows: 4.1% for fT3, 3.4% for fT4, and 5.3% for TSH.

Coronary interventions were performed according to the current practice guidelines and were recorded in digital storage for further analysis. The degree of coronary stenosis was visually estimated by experienced interventional cardiologists. A luminal narrowing > 50% in a major subepicardial vessel (left anterior descending, left circumflex, or right coronary artery) was defined as significant stenosis. Each patient received aspirin plus clopidogrel (loading dose 300 or 600 mg) before or during coronary intervention. Unfractionated heparin 100 U/kg was administered at the beginning of the procedure to keep the activated clotting time > 200 s. The access site for PCI was at the physician’s preference (femoral or radial). The usage of glycoprotein IIb/IIIa (GpIIbIIIa) inhibitors and pre- or post-dilatation after stent implantation of the lesion was at the operator’s discretion. Successful PCI was defined as a < 20% decrease in diameter stenosis and residual stenosis < 5% in diameter with final thrombolysis in myocardial infarction (TIMI) grade 3 flow without any major complications. After stent placement, clopidogrel was administered for 1 month and aspirin was used indefinitely. During routine clinical follow-up, coronary angiography was performed on clinical indications secondarily in patients with stable or unstable angina pectoris. Control coronary angiograms were recorded with Judkins technique and interpreted by two, independent cardiologists who were blinded to the patients’ data. The evaluation of stenosis was conducted using the conventional visual assessment technique. ISR was accepted as narrowing of > 50% in an otherwise normal diameter, including 5 mm proximal and distal to the stent edge, according to the results of control coronary angiographies (19). Intra- and inter-observer variabilities of stent restenosis analysis were minimal in a representative subset of 100 patients. The interpretations of the two investigators on the presence or absence of ISR was 95% and 97%, respectively. Intra-observer variability was assessed by the same investigator. The two readings were concordant for the presence or absence ISR in 98% and 97%, respectively.

Analyses were performed using SPSS 20.0 (SPSS, Inc., Chicago, Illinois). Continuous data were presented as median (minimum-maximum range) or mean ± standard deviation. To test the distribution pattern, the Kolmogorov–Smirnov test was used. The study population was assigned to tertiles based on the preprocedural fT4 level. Comparisons of multiple mean values were conducted by using the Kruskal–Wallis tests or analysis of variance as appropriate. Categorical variables were summarized as percentages and compared with the chi-square test. The effects of different variables on ISR were calculated by univariate analysis for each variable. Variables for which the unadjusted p-value was < 0.10 in the logistic regression analysis were identified as potential risk markers and were included in the full model. We reduced the model using stepwise, multivariate logistic regression analyses and eliminated potential risk markers using likelihood ratio tests. An exploratory evaluation of additional cut points was performed using receiver operating characteristics (ROC) curve analysis. A p-value of < 0.05 was considered statistically significant.

## RESULTS

**Figure 1:**
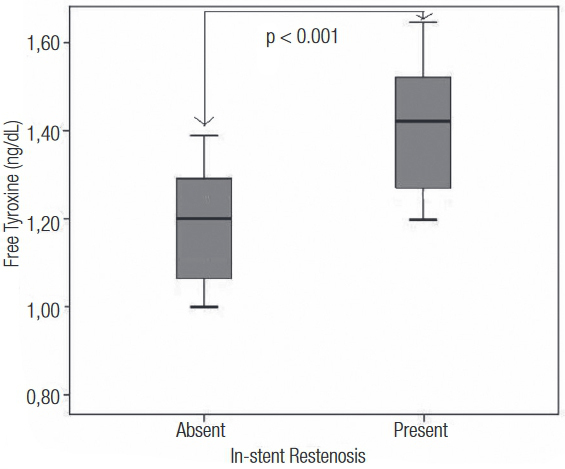
Comparison of serum fT4 levels among patients with and without in-stent restenosis.

A total 705 patients [mean age 60.3 ± 9.3 years, 505 (72%) male] were grouped into tertiles according to the preprocedural fT4 levels. The baseline clinical, laboratory, and angiographic data of the study groups are summarized in Table 1. All diabetic patients included in the study had a diagnosis of type 2 diabetes mellitus. The mean period between the two coronary angiograms for the entire study population was 20.2 ± 4.5 months. While the TSH [median 1.34 (0.95–1.95) vs 1.22 (1.03–1.54), p = 0.48] and fT3 [median 2.96 (2.75–3.18) vs 2.85 (2.60–3.10), p = 0.11] levels were similar between patients with and without ISR, the fT4 levels were significantly higher in patients with ISR [median 1.45 (1.25–1.67) vs 1.20 (1.02–1.38), p < 0.001] (Figure 1). No difference was found in GpIIbIIIa inhibitor use, pre-dilatation rate, or post-dilatation rates among fT4 tertile groups (Table 1) and among the without/with ISR groups [GpIIbIIIa inhibitor use: 36 (7.7%) for without ISR vs 14 (5.9%) for with ISR, p = 0.232; pre-dilatation rate: 27 (8.8%) for without ISR vs 11 (4.6%) for with ISR, p = 0.325; post-dilatation rate: 17 (3.6%) for without ISR vs 7 (2.9%) for with ISR, p = 0.404].

**Table 1:** Baseline characteristics of study sample according to preprocedural free thyroxine tertiles

Variables	Free Thyroxine (ng/dL)			

	Tertile 1 n = 235 1.02 (0.91-1.15)	Tertile 2 n = 235 1.23 (1.13-1.37)	Tertile 3 n = 235 1.49 (1.37-1.68)	p value
Age (years)	60.4 ± 9.1	59.7 ± 9.2	61.1 ± 9.5	0.52
Male gender	178 (77%)	160 (68%)	167 (71%)	0.18
Type 2 DM	53 (23%)	70 (30%)	84 (35%)	0.002
Current smoker	80 (34%)	94 (40%)	115 (49%)	< 0.001
Hypertension	131 (56%)	124 (53%)	135 (57%)	0.58
Cause of stent implantation				
Stable angina pectoris	150 (64%)	146 (68%)	140 (60%)	0.34
Unstable angina pectoris	85 (36%)	89 (38%)	95 (40%)	0.34
No of coronary arteries narrowed				
1 vessel	43 (18%)	56 (24%)	53 (23%)	0.31
2 vessel	192 (82%)	179 (76%)	182 (77%)	0.31
Target coronary artery				
Left anterior descending artery	124 (53%)	100 (43%)	103 (44%)	0.01
Right coronary artery	50 (21%)	83 (35%)	76 (32%)	0.01
Left circumflex artery	61 (26%)	52 (21%)	56 (24%)	0.01
Stent diameter (mm)	3 (2.75-3)	3 (2.5-3)	3 (2.75-3)	0.67
Stent length (mm)	15 (12-18)	15 (13-18)	16 (13-18)	0.31
Periprocedural GpIIbIIIa inhibitor adminisitration	18 (7.7%)	15 (6.4%)	17 (7.2%)	0.86
Pre-dilatation rate	13 (5.5%)	11 (4.7%)	14 (6.0%)	0.82
Post-dilatation rate	8 (3.4%)	7 (3.0%)	9 (3.8%)	0.88
In-hospital medications				
Beta blocker	81%	82%	79%	0.78
ACEi	71%	73%	70%	0.81
CCB	4%	4%	3%	0.84
ARB	5%	6%	5%	0.74
Statins	86%	89%	87%	0.68
LVEF (%)	60.1 ± 3.7	61.0 ± 3.9	59.5 ± 3.4	0.53
Fasting glucose (mg/dL)	104 (92-131)	104 (92-137)	113 (95-144)	0.04
HDL-C (mg/dL)	43 (35-47)	40 (34-46)	35 (30-42)	0.02
LDL-C (mg/dL)	105 (81-134)	107 (83-137)	104 (82-130)	0.73
Triglycerides (mg/dL)	143 (100-207)	140 (100-183)	135 (94-180)	0.18
TSH (μIU/mL)	1.28 (0.12-6.0)	1.40 (0.05-5.90)	1.30 (0.02-5.50)	0.596
Free triiodothyronine (pg/mL)	2.8 (2.6-3.1)	2.9 (2.6-3.2)	2.9 (2.7-3.2)	0.28
Hemoglobin (g/dL)	14.2 ± 2.0	14.0 ± 1.7	13.7 ± 1.6	0.03
Platelet count (×10^9^/L)	251.9 ± 32.2	248.7 ± 34.5	255.6 ± 32.3	0.41
Period between the two CAG (months)	20.9 ± 4.4	20.3 ± 5.2	19.3 ± 4.1	0.17
In-stent restenosis	53 (23%)	82 (35%)	107 (46%)	< 0.001

Data are median (minimum-maximum range), means ± S.D. or n (%).

ACEi: angiotensin converting enzyme inhibitors; ARB: angiotensin receptor blockers; CAG: coronary angiography; CCB: calcium channel blocker; DM: diabetes mellitus; HDL-C: high density lipoprotein cholesterol; LDL-C: low density lipoprotein cholesterol; LVEF: left ventricular ejection fraction.

**Figure 2:**
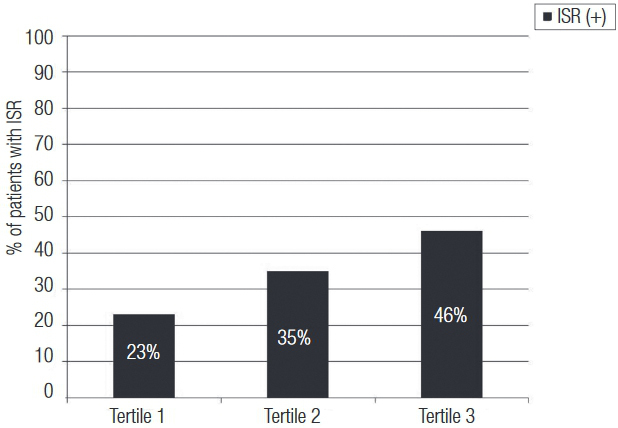
Percentages of the patients developing in-stent restenosis stratified by tertile of preprocedural serum fT4 levels.

The rate of ISR showed an incremental trend from tertile 1 to 3 (23%, 35%, and 46%, p < 0.001, respectively) (Figure 2). Similarly, higher fT4 levels before stent implantation were associated with an increased risk for ISR by logistic regression analysis. Assuming fT4 level as a continuous variable in multiple logistic regression analysis, diabetes mellitus, smoking, HDL-C, stent length, and fT4 levels emerged as independent predictors of ISR (Table 2). When fT4 tertiles were analyzed as a categorical (tertile 1 reference) variable in multiple logistic regression analysis, the relative risk of ISR in the highest tertile was 5.31 (95% CI: 3.44–8.19, p < 0.001) and 2.98 for tertile 2 (95% CI: 1.92–4.63, p < 0.001) as compared to the lowest tertile of the fT4 levels.

**Table 2:** Predictors of in-stent restenosis in univariable and multivariable logistic regression analyses

Variables	Univariable		Multivariable	
	
	OR (95% CI)	p value	OR (95% CI)	p value
Age (years)	1.02 (0.90-1.13)	0.52	-	-
Diabetes mellitus	1.35 (1.12-1.55)	< 0.001	1.21 (1.08-1.33)	< 0.001
Smoking	1.47 (1.08-1.87)	< 0.001	1.87 (1.19-2.55)	0.001
HDL-C (mg/dL)	0.91 (0.86-0.96)	0.001	0.94 (0.89-0.98)	0.02
LDL-C (mg/dL)	1.05 (0.94-1.15)	0.27	-	-
Triglycerides (mg/dL)	1.02 (0.85-1.20)	0.46	-	-
Hemoglobin (g/dL)	1.12 (0.95-1.31)	0.19	-	-
Stent length (mm)	1.33 (1.15-1.51)	0.001	1.30 (1.11-1.48)	0.001
Stent diameter (mm)	1.05 (0.96-1.14)	0.31	-	-
LVEF (%)	1.07 (0.94-1.19)	0.14	-	-
Free thyroxine (ng/dL)	1.17 (1.09-1.25)	0.001	1.05 (1.02-1.08)	0.02
Free triiodothyronine (pg/mL)	1.02 (0.94-1.11)	0.51		
Period between the two CAG (months)	0.93 (0.88-0.97)	0.01	0.97 (0.92-1.03)	0.10

CAG: coronary angiography; CI: confidence interval; HDL-C: high density lipoprotein cholesterol; LDL-C: low density lipoprotein cholesterol; LVEF: left ventricular ejection fraction; OR: odds ratio.

**Figure 3:**
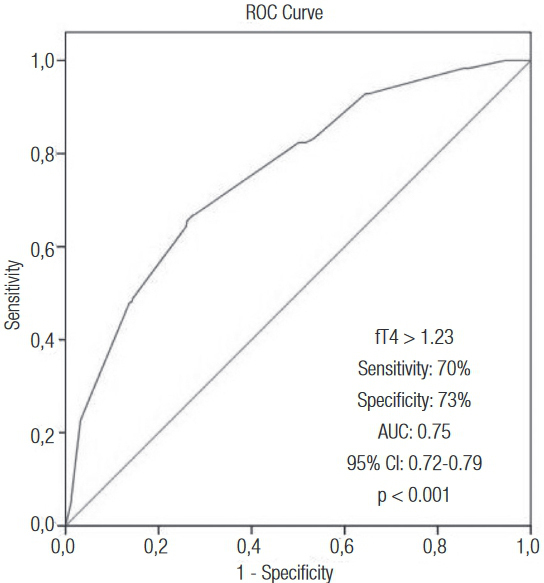
Receiver operating characteristic curve of pre-procedural serum fT4 levels for predicting in-stent restenosis after bare metal stent implantation. AUC: Area under curve; CI: confidence interval.

The ROC curve explored the relation between preprocedural fT4 and ISR. Area under the curve was 0.75 (95% confidence interval 0.72–0.79; p < 0.001). Preprocedural fT4 with a cut-off level of > 1.23 ng/dL well predicted ISR with a sensitivity of 70% and specificity of 73% (Figure 3).

In subgroup analyses, patients with subclinical hyperthyroidism (12/18 patients) displayed a higher rate of ISR during follow-up compared to subclinical hypothyroidism (3/10 patients) and euthyroidism (227/677 patients) (66.7% vs 30% and 33.5%, p < 0.001). In another subgroup analysis that included only diabetic and/or smoking patients, the impact of fT4 on ISR was analyzed. We found that among diabetic patients who did not smoke, the rate of ISR was higher in Tertile 3 [39 (36.4%)] of fT4 compared to Tertile 1 [22 (14.4%)] and Tertile 2 [46 (34.3%)] (p < 0.001). Serum fT4 level was still significantly associated with ISR in patients with diabetes who did not smoke (OR: 3.6, p < 0.001). Among smokers who did not have diabetes, the rate of ISR was higher in Tertile 3 [66 (50.8%)] for fT4 compared to Tertile 1 [22 (11.6%)] and Tertile 2 [52 (31.3%)] (p < 0.001). The serum fT4 level was significantly associated with ISR in non-diabetic smokers (OR: 6.08, p < 0.001). When these same two populations were excluded from the analysis, the rate of ISR was higher in Tertile 3 [18 (35.3%)] for fT4 compared to Tertile 1 [13 (10.9%)] and Tertile 2 [24 (29.6%)] (p < 0.001). However, the serum fT4 level did not reach a high enough statistical significance to show an association with ISR when diabetic and smoking patients were excluded from the analysis.

## DISCUSSION

Three major findings arose from our study: (i) preprocedural serum fT4 is a significant, independent predictor of further ISR in patients with stable or unstable angina pectoris undergoing successful BMS implantation, (ii) patients in the highest tertile of serum fT4 are at greater risk, and preprocedural fT4 > 1.23 ng/dl have a 70% sensitivity and 73% specificity in predicting ISR, and (iii) other well-known predictors of ISR including diabetes mellitus, smoking, lower HDL-C, and increased stent length were also important.

PCI for ischemic coronary heart disease is well-known as a pioneering innovation for interventional cardiologists. However, ISR represents a major nightmare for interventionalists and efforts are ongoing to circumvent this serious problem (15,20). Along with technical issues, several cellular and molecular pathways play a role in coronary wall injury after PCI (14,21). Cellular response to mechanical vascular damage initiated immediately after PCI includes an early phase, consisting of platelet activation and inflammation, followed by an intermediate phase of granulation tissue corresponding to vascular smooth muscle cell migration and proliferation, and a late phase of neointima formation and finally progression to ISR (21). Although the exact etiopathogenesis remains to be fully elucidated, various pre-, peri-, and post-procedural risk factors represent possible actors in the development of ISR (14). The clinical parameter that most consistently increases the risk of ISR is diabetes mellitus (22). The common hypothesis for this effect is that hyperglycemia induces endothelial dysfunction and a proinflammatory state with increased cytokine production (23,24). These pathways enhance neointima formation, which promotes the development of ISR. Although smoking is established as a conventional risk factor for atherosclerosis, there is a possible protective effect associated with smoking that might result in the development of ISR after PCI. The data regarding the impact of smoking on ISR have been contradictory (25-27). Hong and cols. (28) evaluated 840 patients with drug-eluting stent implantation and showed that current smoking was a predictor of ISR in diabetic patients (OR: 1.923, 95% CI: 1.055–4.725). In another study, Ma and cols. (29) also showed that current smoking increases the risk of ISR in ST-segment elevation MI patients undergoing drug-eluting stent implantation. Furthermore, among the lesion-related factors, lesion length and thereby stent length are the most consistent factors that were associated ISR (30). Consistent with previous studies, we have also found that diabetes mellitus, smoking, and stent length are significantly associated with ISR.

The role of THs, particularly fT4, in the pathogenesis of atherosclerosis is unclear and controversial. It has been suggested that THs act both directly and indirectly on atherogenesis by altering vascular tone, modulating macrophage functions, enhancing angiogenesis, and regulating vascular smooth muscle cell proliferation (5-10). Jung and cols. (11) reported that higher serum fT4 levels even within the normal reference range were significantly associated with both the presence and severity of coronary artery disease in 192 patients with stable angina pectoris. In contrast, Auer and cols. (12) showed that higher serum fT4 levels were inversely correlated with the severity of coronary atherosclerosis and higher thyrotropin levels correlated positively. The reasons proposed to underlie the differences in the study results are as follows: i) study populations that are too small to detect an association between subtle changes in fT4 level and coronary atherosclerosis because of the narrow reference range for serum fT4 level; ii) inclusion of heterogeneous patient groups having either stable angina pectoris or acute coronary syndrome. Therefore, to clarify the exact association of THs with coronary atherosclerosis, more specific studies should be designed. The findings of experimental and clinical studies investigating the fT4-atherosclerosis relationship and ISR mechanisms have been coupled, and we have found that high pre-procedural serum fT4 is a powerful and independent predictor of BMS restenosis in patients with stable or unstable angina pectoris. We hypothesize that serum fT4 increases the risk of ISR through enhanced activity of the renin–angiotensin system and proliferation of vascular smooth muscle cells. To the best of our knowledge, this is the first study demonstrating such an association.

Our results raise some clinical implications. The pre-procedural fT4 level may identify patients with a higher risk for ISR with high relevance for clinical routine practice, and close follow-up in those patients is warranted. Furthermore, it is unclear in such a population, at what level or range the serum fT4 should be kept to prevent ISR. Prospective, large-scale studies evaluating the impact of thyroxin replacement or anti-thyroid therapy on ISR should be designed to confirm our study findings and to decide which interval of TH levels are safe for patients undergoing PCI.

Our study should be interpreted within several limitations. First, the study was designed in a retrospective manner representing a single-center experience with only BMS restenosis. Thus, a causal relationship cannot be established between THs and ISR. Second, the definition of ISR was based on visual assessment rather than on a more quantitative and informative intravascular ultrasound or optical coherence tomography. Third, the serum fT4 level was only measured before stent implantation. The levels of serum fT4 have the potential to change over time in an individual patient.

In summary, higher preprocedural serum fT4 is a powerful and independent predictor of BMS restenosis in patients with stable and unstable angina pectoris.
